# From the President’s Desk: Part 3, 2023

**DOI:** 10.4102/safp.v65i1.5791

**Published:** 2023-07-21

**Authors:** Robert J. Mash

**Affiliations:** 1 Division of Family Medicine and Primary Care, Faculty of Medicine and Health Sciences, Stellenbosch University, Cape Town, South Africa

This will be my last letter to you as President of the South African Academy of Family Physicians (SAAFPs). In August, a new Council will take office and they will then vote for new directors of the SAAFP. In this letter, I thought to highlight 10 achievements of the last 6 years, which illustrate how the SAAFP is serving you better as your professional body. If you are reading this and not already a member of the SAAFP (https://saafp.org/about-us-2/membership-2/) then I hope that this will motivate you to join us in strengthening the discipline of family medicine and its contribution to the health system. We need your support to continue serving our discipline.

One of the key focus areas is advocacy for the discipline. In the public sector, we have published a position article (https://safpj.co.za/index.php/safpj/article/view/5473) on the contribution of family physicians to district health services. We have had numerous engagements with government at both national and provincial levels to deliver our message that family physicians are a key intervention to improve service delivery in health centres and district hospitals. Most recently, we met with the Parliamentary Portfolio Committee on Health (https://www.youtube.com/watch?v=YsLb4VY0afE) and Prof. Tasleem Ras led a very successful interaction.

We have also established a Private Sector Forum (https://saafp.org/privatefamilyphysicianforum/) to better advocate for the needs of family physicians. This forum is led by Dr Sheena Mathew and is also writing a position article that can be used in negotiations with medical schemes and private sector institutions. We plan to contract with HealthMan to provide the technical expertise necessary to advocate for the unique practice needs, specialist status and scope of practice. In order to do this, we will need 50 family physicians to pay an additional levy to cover the consultancy fees. If you would like to be part of this initiative, please contact Lucille Boshoff (admin@saafp.org), our administrator.

Another group with specific needs is our newly qualified family physicians. We have established a Next-5 group (https://saafp.org/next5/) to help registrars make the transition to being a family physician during their first 5 years. The group is offering mentoring, webinars, short articles in the *South African Family Practice* journal, and workshops to meet these needs. The group is led by Dr Chantelle van der Bijl – again please contact us if you would like to join this group.

The continuing professional development (CPD) landscape has shifted considerably over the last few years. The need for accreditation of face-to-face events has decreased as virtual events and webinars have mushroomed. Our income from accreditation has substantially decreased. At the same time, our commitment to provide relevant CPD for our members has strengthened – through the *SA Family Practice* journal (https://healthcare-ecpd.co.za/course/index.php?category=219), through eCPD (https://healthcare-ecpd.co.za/mod/page/view.php?id=10548) and by developing bespoke short courses. Our latest short course on Point of Care Ultrasound (POCUS) (https://saafp.org/about-us-2/cpd-accreditation/pocus-accredited-education-program/) is a collaboration with the Global Ultrasound Institute to equip family physicians with these new skills. Our CPD initiatives are led by Prof. Andrew Ross.

This year we also finalised the 4th edition of the South African Family Practice (SAFP) Manual (https://www.vanschaik.com/ebook/6463af1363396/). This edition is an essential resource for all family physicians and covers all the skills required of family physicians across 14 sections and 194 chapters. There is new material on sexual health, POCUS, rehabilitation, community orientated primary care, as well as clinical training. It is an initiative of the SAAFP and edited by Prof. Bob Mash, Prof. Hanneke Brits, Prof. Megan Naidoo and Prof. Tasleem Ras. It is available in both hardcopy and as an e-book.

Our National Education and Training committee coordinates activities across the nine university departments. One of the most successful interventions has been the annual Training of Clinical Trainers course led by Prof. Hanneke Brits. This is raising the standard of workplace-based training and assessment across all programmes. In 2023, we will also introduce accreditation of clinical trainers as a way of further motivating excellence and expertise.

In addition, the SAAFP has taken the lead in coordinating the SCORION e-portfolio (https://saafp.org/scorion-e-portfolio/) for the country and being the first discipline to have a single electronic portfolio of learning. Such an e-portfolio is a vital component of developing workplace-based assessment (WPBA). The National Committee of Deans and Colleges of Medicine are developing WPBA across all specialist disciplines.

The SAAFP has continued to offer an annual National Family Practitioners Conference (https://saafp.org/conferences/2023congress/). As I write this, we are preparing for the 25th conference at The Wanderers in Johannesburg. We are grateful to Dr Keshena Naidoo and her team for organising this conference. The conference is an important opportunity for networking, CPD, learning new skills, addressing topical issues and sharing the latest research. I hope to see you there!

In the last few days, the SAAFP has also submitted a bid to host the WONCA World Conference in 2027 in Cape Town. This global event has only come to Africa once before and by 2027 it will be more than 25 years since it was last here. The event may attract up to 3000 family doctors from around the world. We are actively looking for support from other countries and WONCA regions and hoping that the vote will go our way at the WONCA World Council in October this year.

Finally, we are proud of our *South African Family Practice* journal (https://safpj.co.za/index.php/safpj). Over the last few years, we have successfully switched publishers to AOSIS and appointed a strong team of editors under Prof. Klaus von Pressentin. The journal has done its homework to serve the needs of the discipline through relevant CPD articles, regular columns for the President and Next-5, editorials, open forum articles, Mastering your Fellowship for registrars and consistent publication of original research.

I have been a Director of the SAAFP for more than a decade, and it will be strange to step down and let a new team take the lead. I think we have a solid foundation from which to grow the SAAFP and ensure that we serve our members and contribute to strengthening healthcare for all the people of South Africa. Please ensure you are a member of the SAAFP, enjoy the benefits and participate in strengthening the discipline of family medicine.

